# Long-term potentiation through calcium-mediated N-Cadherin interaction is tightly controlled by the three-dimensional architecture of the synapse

**DOI:** 10.1186/1471-2202-14-S1-P321

**Published:** 2013-07-08

**Authors:** S Grein, S Bunse, E Schuman, G Queisser

**Affiliations:** 1 Goethe Center for Scientific Computing, Frankfurt am Main, Hessen, Germany; 2 Max Planck Institute for Brain Research, Frankfurt am Main, Hessen, Germany

## 

The synaptic cleft is an extracellular domain that is capable of relaying a presynaptically received electrical signal by diffusive neurotransmitters to the postsynaptic membrane. The cleft is trans-synaptically bridged by ring-like shaped clusters of pre- and postsynaptically localized calcium-dependent adhesion proteins of the N-Cadherin type and is possibly the smallest intercircuit in nervous systems [1]. The strength of association between the pre- and postsynaptic membranes can account for synaptic plasticity such as long-term potentiation [2]. Through neuronal activity the intra- and extracellular calcium levels are modulated through calcium exchangers embedded in the pre- and postsynaptic membrane. Variations of the concentration of cleft calcium induces changes in the N-Cadherin-zipper, that in synaptic resting states is rigid and tightly connects the pre- and postsynaptic domain. During synaptic activity calcium concentrations are hypothesized to drop below critical thresholds which leads to loosening of the N-Cadherin connections and subsequently "unzips" the Cadherin-mediated connection. These processes may result in changes in synaptic strength [2]. In order to investigate the calcium-mediated N-Cadherin dynamics at the synaptic cleft, we developed a three-dimensional model including the cleft morphology and all prominent calcium exchangers and corresponding density distributions [3-6]. The necessity for a fully three-dimensional model becomes apparent, when investigating the effects of the spatial architecture of the synapse [7], [8]. Our data show, that the localization of calcium channels with respect to the N-Cadherin ring has substantial effects on the time-scales on which the Cadherin-zipper switches between states, ranging from seconds to minutes. This will have significant effects on synaptic signaling. Furthermore we see, that high-frequency action potential firing can only be relayed to the Calcium/N-Cadherin-system at a synapse under precise spatial synaptic reorganization.
